# A Featured-Based Strategy for Stereovision Matching in Sensors with Fish-Eye Lenses for Forest Environments

**DOI:** 10.3390/s91209468

**Published:** 2009-11-26

**Authors:** Pedro Javier Herrera, Gonzalo Pajares, Maria Guijarro, José J. Ruz, Jesús M. Cruz, Fernando Montes

**Affiliations:** 1 Departamento Arquitectura Computadores y Automática, Facultad de Informática, Universidad Complutense, 28040 Madrid, Spain; E-Mails: jjruz@dacya.ucm.es (J.J.R.); jmcruz@dacya.ucm.es (J.M.C.); 2 Departamento Ingeniería del Software e Inteligencia Artificial, Facultad de Informática, Universidad Complutense, 28040 Madrid, Spain; E-Mail: pajares@fdi.ucm.es; 3 Centro de Estudios Superiores Felipe II, Ingeniería Técnica en informática de Sistemas, 28300 Aranjuez, Madrid, Spain; E-Mail: mguijarro@cesfelipesegundo.com; 4 Departamento de Sistemas y Recursos Forestales, CIFOR-INIA, Ctra. de La Coruña km 7.5, 28040 Madrid, Spain; E-Mail: fernando.montes@upm.es

**Keywords:** stereovision matching, fish-eye lenses, forest image segmentation, feature based

## Abstract

This paper describes a novel feature-based stereovision matching process based on a pair of omnidirectional images in forest stands acquired with a stereovision sensor equipped with fish-eye lenses. The stereo analysis problem consists of the following steps: image acquisition, camera modelling, feature extraction, image matching and depth determination. Once the depths of significant points on the trees are obtained, the growing stock volume can be estimated by considering the geometrical camera modelling, which is the final goal. The key steps are feature extraction and image matching. This paper is devoted solely to these two steps. At a first stage a segmentation process extracts the trunks, which are the regions used as features, where each feature is identified through a set of attributes of properties useful for matching. In the second step the features are matched based on the application of the following four well known matching constraints, *epipolar, similarity, ordering* and *uniqueness*. The combination of the segmentation and matching processes for this specific kind of sensors make the main contribution of the paper. The method is tested with satisfactory results and compared against the human expert criterion.

## 1. Introduction

Forest inventories provide information on which forest management is based. Field surveys, consisting of sample plots situated on a grid, are a technique that has been commonly used in the elaboration of forest inventories for a long time [1,2]. The diameter of trees within these plots is measured. Height, crown height and dimensions, bark thickness and other variables which are more complex to measure are taken in a subsample of trees called second stage trees [3,4]. Taper equations are derived from the second stage trees and calculated for the sample to estimate the growing stock (the volume of wood in a hectare).

In 2005 the Spanish Forest Research Centre (CIFOR) integrated into the National Institute for Agriculture and Food Research and Technology (INIA) patented the MU200501738 forest measurement device. A prototype of the measurement device, adapted for a Nikon® Coolpix® 4500 digital camera with a FC-E8 fish-eye lens and developed by the firm *Railway and Environment Consulting* (Consultoría Ferroviaria y Medioambiental, S.L., C/ Isaac Albéniz, 33, Las Rozas, 28290, Madrid, Spain) for the INIA, was used. This device, located during the image acquisition at a known 3D position in an identifiable geographical direction, allows us to acquire two stereoscopic hemispherical images with parallel optical axes.

Fish eye optics systems can recover 3D information in a large field-of-view around the cameras; in our system this is 183° × 360°. This is an important advantage because it allows one to image the trees in the 3D scene close to the system from the base to the top, unlike in systems equipped with conventional lenses where close objects are partially mapped [5]. Additionally, the direct and diffuse light transmission within all directions of the hemisphere coming from the sky allows one to obtain a well contrasted image as compared to conventional ones [6]. This facilitates the segmentation process described later in Section 2.

Because the trees appear completely imaged, the stereoscopic system allows the calculation of distances from the device to significant points into the trees in the 3D scene, including diameters along the stem, heights and crown dimensions to be measured, as well as determining the position of the trees. These data may be used to obtain precise taper equations, leaf area or volume estimations [7]. As the distance from the device to each tree can be calculated, the density of trees within a determined area can be also surveyed and growing stock; tree density, basal area (the section of stems at 1.30 m height in a hectare) and other interesting variables may be estimated at forest stand level using statistical inference [8].

Moreover, the images constitute a permanent record of the sample point that allows measurement error control and future data mining, which currently requires revisiting the plot. Currently, the above mentioned measurements are manually obtained. An important goal is the automation of the process for data acquisition. Hence, a passive stereovision-based system is a suitable technique for this task, because during the intervention the trees are not affected by the measurement.

According to [9] we can view the classical problem of stereo analysis as consisting of the following steps: image acquisition, camera modelling, feature extraction, image matching and depth determination. These depths allow the computation of the set of measurements mentioned above by considering the geometrical and camera modelling. The key steps are feature extraction and image matching. This paper is devoted solely to these two steps. At a first stage a segmentation process extracts the trunks, which are the regions used as features, where each feature is identified through a set of attributes or properties useful for matching. In the second step the features are matched based on the application of a set of constraints. This matching process tries to identify the corresponding trunks in the two images that are cast by the same physical trunk in the 3-D space. Additionally, in Section 3.1 we give details about the depth determination and how the density in an area and the volume of a tree, among other variables useful for forest analysis, could be estimated.

### 1.1. Constraints Applied in Stereovision Matching

The stereovision sensor provides pairs of images belonging to the same scene captured with two omnidirectional cameras equipped with fish eye lenses. The cameras are separated a given distance among them (base-line). The correspondence problem can be defined in terms of finding pairs of true matches, as explained below, in our approach pairs of regions in two images that are generated by the same physical element in the space. In the proposed method, the regions to be matched are generated by the trunks of the trees. These true matches generally satisfy some constraints [10]: (1) *epipolar*, given a region in an image, the matched region in the second image must lie following the called epipolar line; (2) *similarity*, matched regions must have similar properties or attributes; (3) *ordering*, the relative position between two regions in an image is preserved in the other image for the corresponding matches; (4) *uniqueness*, each region in one image should be matched to a unique region in the other image, although a region could not be matched because of occlusions.

### 1.2. Techniques in Stereovision Matching

A review of the state-of-art in stereovision matching allows us to distinguish two sorts of techniques broadly used in this discipline: *area-based* and *feature-based*. Area-based stereo techniques use correlation between brightness (intensity) patterns in the local neighbourhood of a pixel in one image with brightness patterns in the local neighbourhood of the other image [10]. Feature-based methods use sets of pixels with similar attributes, normally, either pixels belonging to edges [11-13], the corresponding edges themselves [14-16], regions [17,18] or hierarchical approaches [19] where firstly edges or corners are matched and afterwards the regions. In [11] are used regions with the following three specific attributes for matching: area, centroid and angles. They will be used in our proposed approach because of their specific adaptability in the images provided by our sensor.

An important amount of works use the attributes for matching by applying the similarity constraints. In [20] these properties are: area, bounding box and statistical spatial moments. In [21], although under a classification context, first and second statistical moments are used in the HSI colour space; these properties are obtained from the histograms. Also texture descriptors, such as the filters banks are used in [22]. In [23,24] invariant moments have been satisfactorily applied, where it is reported that the feature-based stereovision solution using moment invariants as a metric to find corresponding regions in image pairs, improve the accuracy of the disparity measures. Although in a different context, as it is our forest environment, the idea of accuracy can be useful in our approach. In [25] is proposed a graph-based method to deal with segmentation errors in region-based matching, the nodes in the graph are potential pairs of matches and the arcs have assigned values taking into account a similarity measurement among the regions under matching. In [26] the regions are extracted through a colour based segmentation algorithm and the pixels belonging to the regions are matched obtaining a disparity map, which is then refined by applying cooperative optimization through the adjusting of some parameters in the disparities of the segmented regions. In [27] the colour is also used for segmenting the regions. In [16] vertical lines are used as features in omnidirectional images, a descriptor invariant to rotations is computed. This rotation invariance is useful in our images, as we will see later.

### 1.3. Motivational Research and Contribution

**Figure 1.**
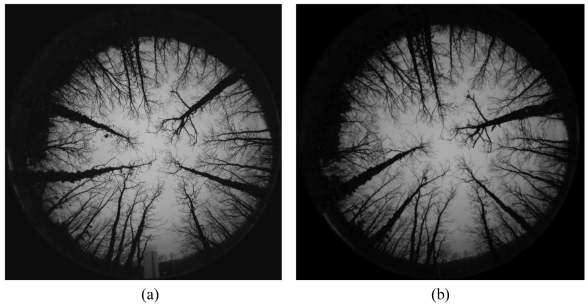
Original omnidirectional images of a stereo pair. **(a)** Left one. **(b)** Right one.

Figure 1 displays a pair of stereoscopic omnidirectional images, which is a representative and illustrative example of the set of sixteen pairs of stereo images used in our experiments. All images were acquired with poor illumination conditions, *i.e.*, on cloudy days without sun or during the dawn or the late afternoon. The sensor is the one described above with a base-line of 1 m. The images resolution is 1,616 × 1,616 pixels, but only the valid central area in the circle containing 2,045,059 pixels is useful.

**Figure 2.**
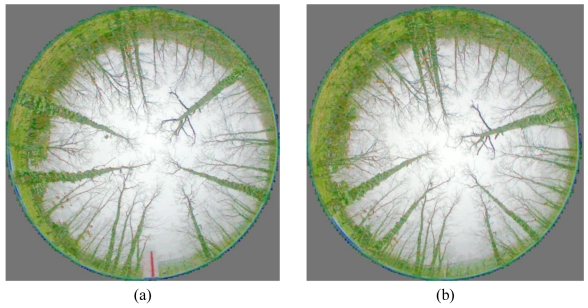
Enhanced omnidirectional images of the stereo pair in Figure 1 by uniform histogram equalization. **(a)** Left one. **(b)** Right one.

The original images are acquired in the RGB colour space. Figure 2 displays both images of Figure 1, but enhanced through uniform histogram equalization [28], applied to the intensity component in the HSI colour model after the transformation from RGB. Now the colour becomes explicit. The kind of images provided by the sensor under the illumination conditions mentioned above, represented by the images in Figures 1 and 2, display a set of specific properties, which are exploited to design our proposed approach. In what follows we discuss about these specific properties oriented towards the choice of the best design strategy as possible.

Our interest is focused on the trunks of the trees because they contain the higher concentration of wood. Therefore, once we have clear that the stereovision matching constraints must be applied, now the problem is to decide if we use area-based or feature-based approaches for matching the trunks. The following is a discussion about which one to use.

#### Area-Based

1. The matching is carried out pixel-by-pixel following the epipolar lines. It does not require a previous knowledge about if the pixel under matching belongs to a trunk or not.
2. The correspondence is established by similarity among the properties of the pixels under matching. The main drawback is that in our images, the trunks and the grass in the soil display similar spectral signatures. They are both dark grey for the images in Figure 1 and green for the images of Figure 2. Hence, in the common parts where soil and trunks are confused the identification of the trunks becomes a difficult task.
3. The part of the image associated to the sky is very homogeneous and the matching pixel by pixel also becomes difficult.
4. Because of the above difficulties, if the correspondence were carried out pixel-by-pixel, after matching we would need to identify the pixels belonging to the trunks.

#### Feature-Based

1. It is the natural choice that a human-based system will use. Indeed, the matching should be carried out by comparing tree-by-tree in both images.
2. The above implies that the human matches the trunks by applying shape similarities between them and also by considering its location in the image based on the epipolar constraint provided by the sensor. The ordering constraint also helps to make the matching.
3. The near radial orientation of the trunks towards the optical centre in the images could be exploited for matching.
4. The main drawback of feature-based in our specific problem, for the automation process, is that the trunks must be identified previously and then a set of properties extracted for their identification.

As one can see, each method has its advantages and disadvantages, so it is unclear which one is the best. An important conclusion concerning both methods is that it is very important that the pixels belonging to the trunks can be univocally identified and isolated. With such purpose and based on the observation of the enhanced images, Figure 2, we have tried to apply texture identification methods for segmenting the trees under different colour spaces following the work in [29]. The colour spaces investigated were the classical ones *RGB, HSI, CIE XYZ*, Lab and Luv, also the log-opponent chromaticity and additionally the red/cyan chromaticity proposed in [29]. From the point of view of textures and based on the results obtained by the different colour spaces, we have applied techniques based on statistical descriptors such as variance and intensity average, both investigated in [28], and also Gabor filters [30]. In [27], a method based on a colour cost function is used for matching, basically for disambiguate false matches. The main problem in our images is that concerning the similarity between the colour and textures in the trunks and those in the soil. After several experiments, we have not achieved satisfactorily the separation of these two kinds of textures through the above approaches. This means that the trunks are not separable through these methods. Moreover, as mentioned before, in [25] a graph-based matching method is proposed for merging and splitting regions that have been incorrectly segmented. This facilitates the posterior matching process. As mentioned before, in our images the main problem is the separation of the trunks from the soil instead of merging those regions. When we try to identify the trunks, we always obtain a unique broad region bordering the outer circumference in the valid image; the splitting of this broad region, based on intensity or texture dissimilarities, becomes a very difficult task. Nevertheless, in our proposed approach we are able to apply the splitting concept but under geometrical considerations, as we will see below, with satisfactory results. The method proposed in [26] requires the computation of an initial dense disparity map, which is later refined by fitting a plane over the segmented regions. In our approach, an important problem is that concerned with the segmentation of the regions and the computation of the disparity map.

Due to the above handicaps and because of the spatial geometrical distribution of the trees in each stereo-pair, we have finally designed a new feature-based approach that can cope with the problem of separating the trunks from the soil. So, in the part of the image where the textures belonging to the trunks can be easily distinguished from those belonging to the sky, we separate them by considering intensity dissimilarities and in the part where the textures belonging to the trunks and soil are indistinguishable we estimate the trunk position guided by the geometrical knowledge derived from the first part. After the segmentation of the trunks, a set of properties is obtained. Then the application of the matching constraints, involving similarities between properties and geometrical relations based on the sensor geometry, allows the matching of the trunks in both images of the stereo pair. In summary, the full stereovision matching process involves two main steps, namely: trunk segmentation and correspondence of the trunks.

**Figure 3.**
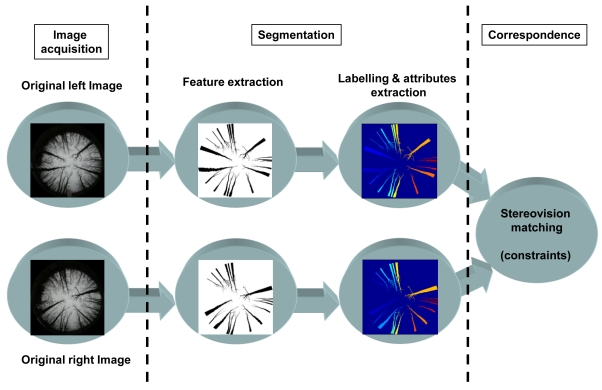
Scheme of the stereovision matching process.

Figure 3 displays the architecture of the proposed full process. Two images are available after their acquisition by the stereovision sensor. Unlike the classical stereovision sensors based on parallel optical axes, where the left and right images are captured by each camera located on the left and right positions, in our omnidirectional image based sensor no distinction can be made between left and right images. Nevertheless, without loss of generality, one image in this kind of sensors is called the left image and the second the right one.

Once the images are available, the full stereovision matching process consists of the following two main steps:

1. *Segmentation*: both images are processed so that a set of regions, corresponding to the trunks, are extracted and then labelled. Each region is identified by a set of attributes, including the Hu invariant moments [28], the position and orientation of the centroid and the area.
2. *Correspondence*: based on these attributes and applying the stereovision matching constraints, where the sensor geometry is specifically considered, the matching between the regions in both images can be established.

The main contribution of this paper is the design of the specific segmentation process combined with the correspondence one. Both define the full stereovision matching procedure for this kind of sensors in the type of images considered. The performance of the proposed automatic approach is compared favourably against the criteria of the human expert, which processes the images based on his expertise, but manually. The proposed strategy is limited to the type of images described above, basically acquired with poor illumination and high contrast between the sky and the trunks in the central part. Under different conditions other strategies must be applied. Indeed, in [31] an area-based matching strategy is used for pinewoods.

### 1.4. Paper Organization

This paper is organized as follows. In Section 2 we describe the procedures applied for the image segmentation oriented to the identification of regions describing the trunks. Section 3 describes the design of the correspondence process by applying the epipolar, similarity, ordering and uniqueness constraints. Section 4 contains the results obtained by the proposed stereovision matching approach under the criteria of the expert human. A discussion about them is also included. Section 5 presents the conclusions and future work plans.

## 2. Segmentation Process

As mentioned before, the goal of the segmentation is to extract automatically the regions associated to the trunks and their properties, so that these regions can be matched in both images.

Based on the observation of the images processed, represented by the one in Figure 1 or equivalently Figure 2, the following details can be inferred:

1. The Charge Coupled Device (CCD), in both cameras, is rectangular, but the projection of the scene through the fish-eye lenses result on a circular area of the scene, which is the valid image to be processed.
2. In the central part of the image, until a given level, the sky and the trunks are easily distinguished because of its contrast. Unfortunately this does not occur in the outer part of the valid circumference because the trunks and the grass in the soil display both similar spectral signatures in the RGB colour space. They are dark gray in the image in Figure 1 and green in the enhanced image of Figure 2.
3. The trunks display an orientation towards the centre; this means that in the 3D scene they are near vertical. Nevertheless, there are some of them that are not exactly vertical and even capricious forms could appear. This must be taken into account because it impedes us to apply exactly the geometrical radial property during the segmentation.
4. The trees are clean of leaves in the branches, this facilitates their identification.
5. Because the cameras in the stereovision sensor are separated by the base-line, 1 m in our sensor, the same tree is not located in the same spatial position in both images. A relative displacement, measured in degrees of angle, appears between corresponding matches. This displacement is greater for the trees near the sensor than for those who are far.
6. Depending on the position of each tree with respect each camera, the images of the trees appear under different sizes, affecting the area of the imaged trunk in both images. A tree near the left camera appears with an area in the left image greater than the area in the right one and vice versa.

**Figure 4.**
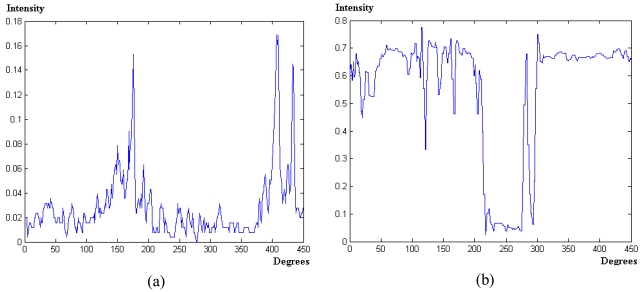
Intensity profiles. **(a)** The circumference cross a region where the trunks and the soil cannot be distinguished. **(b)** Low and high intensity levels. The first are associated to trunks and the second to the sky. Intensities vary from 0 to 1.

**Figure 5.**
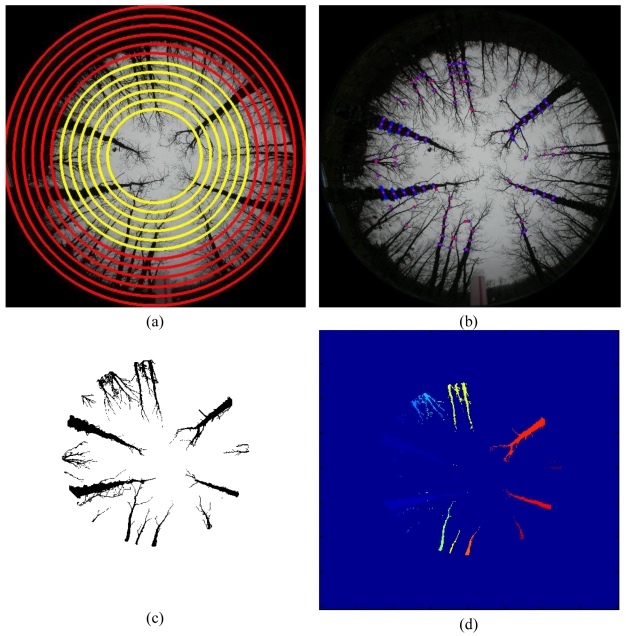
**(a)** Yellow and red drawn circumferences. **(b)** Homogeneous regions drawn in blue and seeds in red. **(c)** Resulting image obtained with the *region growing* process. **(d)** Resulting image obtained with the *labelling* process.

**Figure 6.**
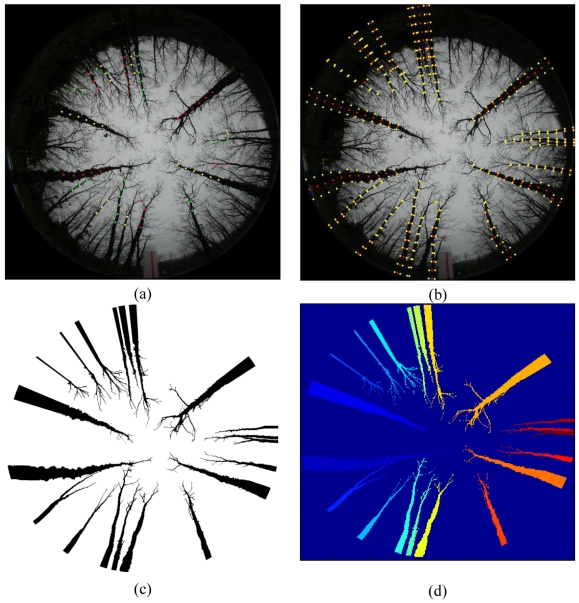
**(a)** Seeds classified depending on the region to which they belong. By clarity we can see the classified seeds painted with the same colour. **(b)** Resulting image obtained with the steps: *prediction* and *correction*. The seeds are drawn in red. Each region is delimited with yellow points taking into account the associated yellow circumferences. **(c)** Resulting image obtained with the second *region growing* process. **(d)** Resulting image obtained after the *relabeling* process.

Now we are able to define the segmentation process based on the following steps according to the above observations:

**Step 1** *Valid image*: each CCD has 1,616 × 1,616 pixels in width and height dimensions respectively. Considering the origin of coordinates in the left bottom corner, the centre of the image is located in the coordinates (808, 808). The radius *R* of the valid image from the centre is 808 pixels. So, during the process only the image region inside the area limited by the given radius is to be considered. Moreover, we work with the intensity image *I* in the *HSI* colour space obtained after the transformation from *RGB* to *HSI*. This is because, as mentioned before, we have not achieved satisfactory results with the studied colour spaces and the image *I* contains the spectral information of the three R, G and B spectral channels. The region growing process, applied later, works better in the original image, Figure 1, than in the enhanced one, Figure 2, because of the similarity on the intensity values in the original one. This justifies the use of the original images instead of the enhanced ones. Later, in section 4, we give details about the protocol for measuring a sample plot in the forest, where the sensor is located in the centre of a circle with radius ranging from 5 m to 25 m. Hence, only the trunks inside circles with radius below 25 m are of interest and they are imaged with an appropriate area for their treatment, the remainder ones are projected with small areas and their treatments become complicated.
**Step 2** *Concentric circumferences*: we draw concentric circumferences on the original image, starting with a radius *r* = 250 pixels with increases of 50 pixels until *r* = *R*. For each circumference, we obtain the intensity profile. There are two main types of circumferences, namely: those in zones where all trunks are well contrasted with respect the background and those in zones where the background and the trunks get confused. Figure 5(*a*) displays both types of zones, the first ones are drawn in yellow and the second ones in red. As one can see, the yellow circumferences cross areas with the trunks over the sky and the red ones cross zones where the trunks and the soil appear with similar intensity levels.
**Step 3** *Intensity profiles and detection of crossed dark regions*: following the circumference paths, we draw the associated intensity profile for each one. Figure 4 displays two intensity profiles covering a range of 45° in this representation from 135° to 180°. In the profile appear low and high intensity levels. The low ones are associated to trunks or soil and the high ones to the sky. Based on the above, if large dark areas appear in the profile, this means that the circumference cross a region where the trunks and the soil cannot be distinguished and this circumference is labelled as red. This occurs in the Figure 4(a) which represents low intensity values ranging from 0 to 0.18 over a range of [0,1] *i.e.*, a large dark area. If no large dark areas are identified, the circumference is labelled as yellow. On the contrary if a relative small dark area appears limited by two clear areas, it represents a trunk; Figure 4(b).
**Step 4** *Putting seeds in the trunks*: considering the yellow circumferences, we are able to detect the trunks positions crossed by them, which are dark homogeneous regions in the profile limited by clear zones, Figure 4(b). This allows choosing a pixel for each dark homogeneous region; such pixel is considered a seed. Also, because we know the transition from clear to clear crossing a dark homogeneous region, we obtain the average intensity value and standard deviation for it. In summary, from each dark homogeneous region in a yellow circumference, we select a seed and obtain its average intensity value and standard deviation.
**Step 5** *Region filtering*: we are only interested in specific dark regions, considered as those that represent trunks of interest. The process of selecting them is as follows, Figure 5(b).
  - We consider only those dark regions in the profile where the intersection with yellow circumferences produces a line with more than *T*_1_ pixels. This is to guarantee that the trunk analyzed is wide enough. Its justification is because we assume that this kind of trunks belong to the area of interest under analysis, *i.e.*, to the circle with radius lesser than 25m.
  - Also, based on the yellow circumferences, we only consider the regions with intensity levels less than *T*_2_ because we are dealing with dark homogeneous regions (trunks).
  - Considering the outer yellow circumference *c_i_*, we select only dark regions whose intersection with this circumference gives a line with a number of pixels lower than *T*_3_. The maximum value in pixels of all lines of intersection is $t_{\max}^{i}<T_{3}$. Then for the next yellow circumference towards the centre of the image, *c*_*i*+1_, *T*_3_ is now set to $t_{\max}^{i}$, which is the value used when the next circumference is processed and so on until the inner yellow circumference is reached. This is justified because the thickness of the trunks always diminishes towards the centre.
  In this work, *T*_1_, *T*_2_ and *T*_3_ are set to 10, 0.3 and 120 respectively, after experimentation.
**Step 6** *Region growing*: this process is based on the procedure described in [28], we start in the outer yellow circumference by selecting the seed pixels obtained in this circumference. From these seed points we append to each seed those neighbouring pixels that have a similar intensity value than the seed. The similarity is measured as the difference between the intensity value of the pixel under consideration and the mean value in the zone where the seed belongs to, they do not differ more than the standard deviation computed in step 4 for that zone. The region growing ends when no more similar neighbouring pixels are found for that seed between this circumference and the centre of the image. This allows obtaining a set of regions as displayed in Figure 5(c).
**Step 7** *Labelling*: before the labelling, an opening morphologic operation is applied. The aim is to break joined links, to avoid that some branches of the trees overlapping other branches or trunks lead to label two trees or trunks as a unique region. The structural element during the opening is the classical 3 × 3 matrix of ones because it is symmetric operating in all spatial directions. The regions extracted during the previous *region growing* are labelled following the procedure described in [32]. Figure 5(d) displays this step.
**Step 8** *Regions and seeds association*: for each one of the seeds in the outer yellow circumference, we make to correspond to each seed its region identified before. It is possible that more than one seed turns out to be belonging to the same region. If this occurs, we create new regions, so that finally we obtain the same number of regions than seeds. After this step, each region has assigned a unique seed.
**Step 9** *Seeds association*: we check for the other seeds in the remainder yellow circumferences. If a seed fulfils that it is the nearest in terms of pixel distance and its angle in degrees the most similar to the angle of the previously checked seed, then it belongs to the same region that the seed checked previously, which is the reference. The angle in degrees is the *θ* value in polar coordinates (*ρ,θ*) with respect the seed location in Cartesian coordinates (*x,y*). This process allows establishing correspondences among the seeds of the different yellow circumferences depending on the region to which they belong, Figure 6(a), *i.e.*, to identify seeds that belong probably to the same region (trunk). We compute the average orientation, *s̄*, for all seeds belonging to the same region identified according to the process described in this point.
**Step 10** *Estimation of the seeds locations in the red circumferences*: it consists of three sub steps, *prediction, correction* and *measurement*:
  - *Prediction*: the pixels belonging to a trunk crossed by a red circumference must have identical orientation, in degrees, that the seed in the outer yellow circumference crossing the same trunk. So, we obtain the seeds in red circumferences fulfilling this and starting from the inner one.
  - *Correction*: since there are trunks that are not aligned towards the centre, the prediction can introduce mistakes. An offset value is applied to this location, which is exactly *s̄*, computed in step 9, Figure 6(b).
  - *Measurement*: after the offset correction, we verify if the estimated seed in each red circumference belongs to a trunk. This is only possible if the red circumference crosses a region with low intensity values limited by zones with high intensity values and the estimated seed location is inside the region with low values. With this, we assume that the seed belongs to the same trunk that the seed in the yellow circumference. Because of the contrast in the intensity profile for the red region in the specific trunk, we can measure the exact seed location in the central part of the low intensity region. The estimated seed location is replaced by the measured one and used for estimating the next seed location in the next red circumference. If the profile does not display low and high intensity values, no measurements can be taken and the next seed location is the previously estimated by *prediction* and *correction*.
**Step 11** *New* r*egion growing*: starting on the outer yellow circumference, we apply again a new region growing process as the one described in the step 6, but now controlled by several iterations (so many iterations as red circumferences). For each iteration, the region growing has its upper limit given by the radius of the nearest red circumference. Once the outer red circumference is reached, *i.e.*, maximum number of iterations, the region growing ends; at this moment an opening morphologic operation is applied trying to break links between regions (trunks) which could be still joined. The structural element used for the opening is the same that the one used in step 7. Figure 6(c) displays this step.
**Step 12** *Relabeling*: this process is similar to the one described in step 7. We re-label each one of the regions that have appeared after the region growing process in step 11, Figure 6(d).
**Step 13** *Attributes extraction*: once all regions have been relabelled, for each region we extract the following attributes: area (number of pixels), centroid (*xy*-averaged pixel positions in the region), angles in degrees of each centroid and the seven Hu invariant moments [28].

## 3. Correspondence Process

Once the segmentation process has finished, we have available a set of regions identifying trunks in both images of the stereo pair. Each region has associated the above mentioned attributes (area, centroid, angles and Hu invariant moments).

As mentioned in Section 1.1, in stereovision matching there are a set of constraints that are generally applied for solving the matching problem. In this work we have applied: *epipolar, similarity, ordering and uniqueness*.

Now, we use conveniently the attributes according to the requirements of each constraint. In what follows, Sections 3.1 to 3.3, we explain how the correspondence process is carried out.

### 3.1. Epipolar: Centroid

**Figure 7.**
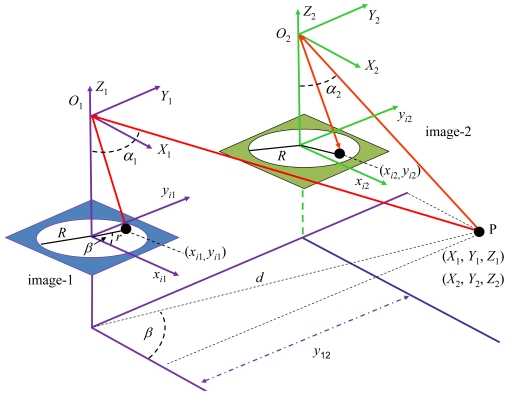
Geometric projections and relations for the fish-eye based stereo vision system.

The centroid of each region is used under the epipolar constraint, as a guide for matching as we explain below. Based on the sensor geometry, the epipolar lines can be established as described below. Figure 7 displays the stereo vision system geometry [5]. The 3D object point *P* with world coordinates with respect to the systems (*X*_1_, *Y*_1_, *Z*_1_) and (*X*_2_, *Y*_2_, *Z*_2_) is imaged as (*x*_*i*1_, *y*_*i*1_) and (*x_i_*_2_, *y_i_*_2_) in image-1 and image-2 respectively in coordinates of the image system; *α*_1_ and *α*_2_ are the angles of incidence of the rays from *P*; *y*_12_ is the base-line measuring the distance between the optical axes in both cameras along the *y*-axes; *r* is the distance between image point and optical axis; *R* is the image radius, identical in both images.

According to [33], the following geometrical relations can be established:

(1) $$r=\sqrt{x_{i1}^{2}+y_{i1}^{2}};\alpha_{1}=\frac{\pi r}{2R};\beta =tg^{-1}\left(y_{i1}/x_{i1}\right)$$

Now the problem is that the 3D world coordinates (*X*_1_, *Y*_1_, *Z*_1_) are unknown. They can be estimated by varying the distance *d* as follows:

(2) $$X_{1}=d\cos\beta ;Y_{1}=d\sin\beta ;Z_{1}=\sqrt{X_{1}^{2}+Y_{1}^{2}}/\tan\alpha_{1}$$

From (2) we transform the world coordinates in the system *O*_1_*X*_1_*Y*_1_*Z*_1_ to the world coordinates in the system *O*_2_*X*_2_*Y*_2_*Z*_2_ taking into account the base-line as follows:

(3) $$X_{2}=X_{1};\;Y_{2}=Y_{1}+y_{12};\;Z_{2}=Z_{1}$$

Assuming no lenses radial distortion, we can find the imaged coordinates of the 3D point in image-2 as [33]:

(4) $$\begin{array}{ll} x_{i2}=\frac{2R\arctan\left(\sqrt{X_{2}^{2}+Y_{2}^{2}}/Z_{2}\right)}{\pi \sqrt{{\left(Y_{2}/X_{2}\right)}^{2}+1}}; & y_{i2}=\frac{2R\arctan\left(\sqrt{X_{2}^{2}+Y_{2}^{2}}/Z_{2}\right)}{\pi \sqrt{{\left(X_{2}/Y_{2}\right)}^{2}+1}} \end{array}$$

**Figure 8.**
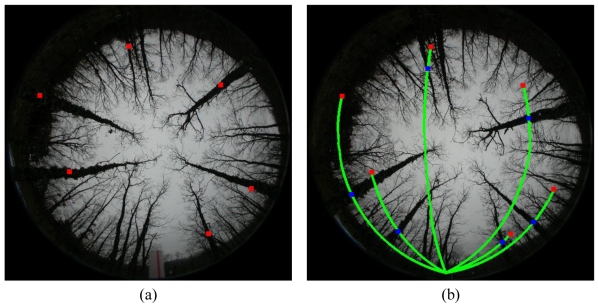
**(a)** Left image marked with six squares. **(b)** Epipolar lines in the right image generated from the locations in the left image marked with the squares.

Because of the system geometry, the epipolar lines are not concentric circumferences and this fact is considered for matching. Figure 8(b) displays six epipolar lines in the right image, which have been generated by the six pixels located at the positions marked with the squares; they are their equivalent locations in the left image [Figure 8(a)].

Using only a camera, we capture a unique image and each 3D point belonging to the line $\bar{O_{1}P}$, is imaged in (*x*_*i*1_, *y*_*i*1_). So, the 3D coordinates with a unique camera cannot be obtained. When we try to match the imaged point (*x*_*i*1_, *y*_*i*1_) into the image-2 we follow the epipolar line, *i.e.*, the projection of $\bar{O_{1}P}$ over the image-2. This is equivalent to vary the parameter *d* in the 3-D space. So, given the imaged point (*x*_*i*1_, *y*_*i*1_) in the image-1 (left) and following the epipolar line, we obtain a list of *m* potential corresponding candidates represented by (*x_i_*_2_, *y_i_*_2_) in the image-2 (right). The best match is associated to a distance *d* for the 3D point in the scene, which is computed from the stereo vision system. Hence, for each *d* we obtain a specific (*x_i_*_2_, *y_i_*_2_), so that when it is matched with (*x*_*i*1_, *y*_*i*1_) *d* is the distance for the point *P* from the sensor. Our matching strategy identifies correspondences between regions or simplifying correspondences between two image pixels (*x*_*i*1_, *y*_*i*1_) and (*x_i_*_2_, *y_i_*_2_). Based on this correspondence we start from Equation (1) and then we give values to the variable *d* until the values of (*x_i_*_2_, *y_i_*_2_) obtained through Equation (4) are equal or as close as possible to the ones obtained by the stereovision matching process. So, we obtain the value of *d* that best fits both pixels, it is from the sensor to the 3D point *P*, Figure 7. A distance from the sensor to the centroid of a region determines the distance to the tree it represents; with this distance we make the decision about its inclusion in the sample plot for tree density and basal area estimation. From the central angle covered by the trunk width and the distance from the sensor, the diameter of the tree can be measured at different heights on the stem. Also we can compute distances from the sensor to points in the base and the top of a tree, with these distances and using the angles of projection *α*_1_ obtained with Equation (1) for these points, we can compute the height of the tree by applying trigonometric rules such as the cosine theorem. The above reasoning is also applicable for computing distances to significant points; this allows to measure other variables described in the introduction.

Based on the above, given a red square in the left image, following the epipolar line in the south direction we will find the corresponding matching, Figure 8(b). This implies that given a centroid of a region in the left image its corresponding matching in the right image will be probably in the epipolar line. Because the sensor could introduce errors due to wrong calibration, we have considered an offset out of the epipolar lines quantified as 10 pixels in distance. Moreover, in the epipolar line, the corresponding centroids are separated in a certain angle, as we can see in Figure 8(b) expressed by the red and blue squares. After experimentation with the set of images tested, the maximum separation found in degrees has been quantified in 22°. Obviously, the above is applicable considering the left image as the reference, but if we consider the right one as the reference, the search for correspondences in the left one is made in the opposite direction. Based on the work of [34], given a centroid of a region in the left image we search for its corresponding centroid in the right one following the epipolar lines drawn in Figure 8(b) and then given a centroid in the right image, we search in the reverse sense in the left one, also following the epipolar lines.

### 3.2. Similarity: Areas and Hu Moments

As mentioned before, each region in both, left and right images, of the stereo pair has its own set of properties. The Hu moments are invariant to translations, rotations and scale change. Of particular interest is the invariance to rotations because the trunks appear rotated in the right image with respect the left one and vice-versa. This is an important advantage of these moments for the matching.

On the contrary, the scale change represents a disadvantage because a large region in one image could be matched with a small one in the other image, both with similar form and aspect.

Due to the sensor geometry, a tree close to a camera is imaged under an area greater than its area in the other camera. This implies that the correct matches generally display different area values.

To overcome the above problems and simultaneously exploit all available information provided by the sensor geometry, we define the following procedure. Before describing it, let us introduce some definitions.

Say {*L*_1_, *L*_2_, …, *L_NL_*} and {*R*_1_, *R*_2_, …, *R_NR_*}two sets of feature descriptors representing the segmented and labelled regions in the left and right images respectively of a stereo pair supplied by the sensor. *NL* and *NR* are the number of features in the left and right image respectively.

Each feature *L_i_* contains: the area (*A_i_*), the centroid (*x_i_, y_i_*) and a vector with the seven Hu invariant moments ${\text{h}}_{i}\equiv \left\{\phi_{1}^{i},\phi_{2}^{i},\ldots ,\phi_{7}^{i}\right\}\in {ℜ}^{7}$, *i.e., L_i_* ≡ {*A_i_*, (*x_i_, y_i_*), ***h**_i_*}. Similarly, for R_j_ ≡ {A_j_, (*x_j_, y_j_*), ***h**_j_*} where as before ${\text{h}}_{j}\equiv \left\{\phi_{1}^{j},\phi_{2}^{j},\ldots ,\phi_{7}^{j}\right\}\in {ℜ}^{7}$. Because the seven moments range in different scales, we map linearly each Hu moment to range in the interval [0,1] as follows:

(5) $$\Phi_{k}^{h}=\frac{\phi_{k}^{h}-m_{k}}{M_{k}-m_{k}};h=i,j\;\text{and}\;k=1,2,\ldots ,7$$

where $M_{k}=\max\left\{\phi_{k}^{i},\phi_{k}^{j}\right\}$ and $m_{k}=\min\left\{\phi_{k}^{i},\phi_{k}^{j}\right\};\forall i,j$.

Then, say:

(6) $${\text{D}}_{k}^{i}=\left\{\left|\Phi_{k}^{i}-\Phi_{k}^{j}\right|,\;j=1,2,\ldots ,NR,\;k=1,2,\ldots ,7\right\}$$

is the set of all distances between a given component $\Phi_{k}^{i}$ and all $\Phi_{k}^{j}$, *j* = 1, 2, …, *NR*. Instead of computing a distance for matching between the vectors ***h**_i_* and all ***h**_j_*, we have preferred to compute the individual distances ${\text{D}}_{k}^{i}$. This is justified because each moment normally contributes in a different fashion as an attribute for the matching between features *L_i_* and *R_j_*, so when using distances between those vectors, such as the Euclidean one, the individual contributions could be masked.

Now the problem is: given a feature *L_i_* in the left image, which its matched feature *R_j_* is in the right image? Following the work of Scaramuzza et al. [16] we establish the following conditions, derived from the Hu invariant moments that must be fulfilled. So, *L_i_* and *R_j_* match, based on $\Phi_{k}^{i}$ and $\Phi_{k}^{j}$ if:

- $d_{k}^{ij}=\min\left\{{\text{D}}_{k}^{i}\right\}<T_{1}$. This means that the minimum distance is indeed obtained for $\Phi_{k}^{i}$ and $\Phi_{k}^{j}$ and it is smaller than a fixed threshold *T*_1_, *i.e.*, only small distances are accepted. *T*_1_ is set to 0.3 after trial and error.
- $d_{k}^{ij}=\min\left\{{\text{D}}_{k}^{i}\right\}<T_{2}\langle {\text{D}}_{k}^{i}\rangle$. This means that the minimum distance is smaller enough than the mean of the distances from all other distances between features, where $\langle {\text{D}}_{k}^{i}\rangle$ is the mean value of ${\text{D}}_{k}^{i}$ and *T*_2_ is a threshold ranging from 0 to 1. It has been set to 0.5 in our experiments, because we have verified that it suffices as in [16].
- the rate between $d_{k}^{ij}$ and the second minimum distance $d_{k}^{ih}=\min\left\{{\text{D}}_{k}^{i}\right\}$ with *h* = 1, 2, …, *NR* and *h* ≠ *j*, is smaller than a threshold *T*_3_, set to 0.3 in our experiments. This guarantees that a gap between the absolute minimum distance and the second one exists.
  As mentioned before, because the sensor is built with two cameras, with a given base-line, the same tree in the 3D scene can be imaged under different areas in both images. This issue has been addressed in [35] where an exhaustive study is made about the different shapes in the images of the same 3D surface in conventional sensors. Here, it is stated that there is not a unique correspondence between a pixel in one image an other pixel in the other image. This is an important reason for using regions as a feature-based approach instead of area-based because the above problem does not occur. Now two trunks, which are true matches, one belonging to an image and the other to the second, can display different areas. Therefore, we formulate the following condition for matching two regions by considering both areas:
- The areas *A_i_* and *A_j_* do not differ between them more than the 33%.

### 3.3. Ordering: Angles

The relative position between two regions in an image is preserved in the other one for the corresponding matches. The ordering constraint application is limited to regions with similar heights and areas in the same image and also if the areas overpass a threshold *T*_4_ set to 6,400 in this paper. The similarity is defined, as above, in the point D, *i.e.*, with relative differences below the 33%. This tries to avoid violations of this constraint based on closeness and remoteness relations of the trunks with respect the sensor in the 3D scene (see Section 4.2 for details in the stereo pair displayed), which are applicable to the remainder stereo pairs analyzed.

Then, given an order for the trunks in the left image, this constraint assumes that the same order is kept in the right one for the respective corresponding trunks and vice versa, Figure 1a,b.

To apply this constraint, we obtain the coordinates of the centroid of each region to calculate its orientation in degrees.

**Figure 9.**
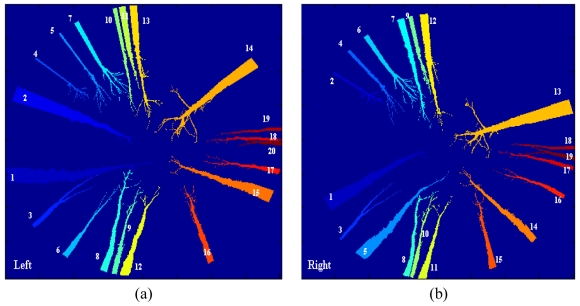
Labelling regions: **(a)** left image, **(b)** right image. Each region appears identified by a unique number.

The following pedagogical clarifies this. In Figure 9a,b, the trunk labelled as 2 in the left image matches to the labelled as 1 in the right one. The region labelled as 1 in the left image matches to the labelled as 5 in the right one. Following the clockwise sense region 1 is found before region 2 in the left image and their corresponding matches preserve this order, *i.e.*, 5 is found before 1 in the right image. The criterion “found before” is established by considering the angle orientation of the respective centroids.

### 3.4. Summary of the Full Correspondence Process

Based on the above, the full matching process can be summarized as follows:

#### Correspondence Left to Right

For each region *L_i_* in the left image we search for candidates *R_j_* in the right image, according to the steps defined below.

1. Apply *epipolar* constraint: we only consider potential matches of *L_i_* those *R_j_* regions that fulfil the epipolarity, as defined in the subsection 3.1. After this step, *L_i_* has as potential candidates a list *l_i_* of *n* regions in the right image, say *l_i_* ≡ *L_i_* → {*R_j_*_1_, .., *R_jn_*}, where *j*1, *jn* ∈ {1, …, *NR*}.
2. Apply the condition D given in Section 3.2 to the list *l_i_*. Exclude from *l_i_* those candidates that do not fulfil such condition D.
3. Apply conditions A to C given in Section 3.2 to the current list *l_i_*. For each pair (*L_i_, R_jn_*) obtained from *l_i_*, determine if *L_i_* and *R_jn_* match based on the *k^th^* Hu moment according to such conditions A to C. Define *lk* as the number of these individual matches.

#### Correspondence Right to Left

4. For each region *R_j_* in the right image we search for candidates *L_j_* in the left image, following similar steps to the previous ones. Now a list *r_j_* of candidates is built and a number *rk* of individual matches is obtained according to the Hu invariant moments. The epipolar constraint is applied following the same lines than those used for Left to Right but in the reverse sense.

#### Final Decision: Simple majority and Uniqueness

5. We say that *L_i_* matches with *R_j_*, iif *lk* + *rk* > *U*, where *U* has been set to 7 in our experiments. This value has been fixed taking into account that the maximum value that the sum *lk* + *rk* can achieve is 14, *i.e.*, a value greater than 7 represents the majority.
6. If the matching between *L_i_* and *R_j_* is unambiguous the correspondence between both features is solved; otherwise in the ambiguous case, where the above condition is fulfilled by more than one match, then we apply the ordering constraint based on the unambiguous correspondences which have been already solved. This implies the application of both *ordering* and *uniqueness* constraints simultaneously.

## 4. Results

The final goal is to obtain measurements about the trees in sample plots, typically circular shaped with radius ranging from 5 to 25 m, located in the forest stand at distances ranging from 100 to 1,000 m from each other. With such purpose, the stereovision sensor is located at the centre of the plot. The images contain trees belonging to the sample plots and also trees out of the sample plots. Only the first ones are of interest. Although some trees out of the plots are processed by the reasons given in Section 4.1, they are of no interest.

The centres of the plots are known 3D geographical positions previously obtained via GPS. Moreover, as mentioned during the introduction, the sensor is positioned under the identifiable geographical direction normally the left camera oriented towards the North and the right one toward the South and both with the base-line of 1*m*. This allows that different measurements spaced in the time, probably years, are obtained under the same criteria. This allows one to compare the values of the variables measured in different times and derive annual increments. The tests have been carried out with sixteen pairs of stereo images. The pair of images displayed in this work is a representative example of them including all relevant aspects described during the introduction, which characterize this kind of sensed images. Because our proposal consists of two phases, some considerations can be made about each one.

### 4.1. Segmentation

Figures 9a,b displays the regions extracted by the segmentation process. Each region appears labelled with a unique label. The number near of the regions identifies each label. This number is represented as a colour in a scale ranging from 1 to 14, where 1 is blue and 14 orange. This representation is only for a best visualization of the regions.

From Figure 9, the following conclusions can be inferred:

1. The regions have been well separated, even if there were regions very near among them. This occurs with the regions 10 and 11 or 18 and 20 in the left image and also with the regions 8 and 10 in the right one.
2. The procedure is able to extract regions corresponding to trunks, which are relatively far from the sensor, *i.e.*, out of the area of the sample plot, which is the area of interest. This occurs with the regions labelled as 4, 5, 18, 19 and 20 in the left image and 2, 17, 18 and 19 in the right image. Although such regions are out of the interest, we have preferred include them for matching, because in the future perhaps the sensed area could be extended to an area greater than 25 m and also to verify the robustness of the correspondence process. Its exclusion is an easy task because they all fulfil that their areas are below a value of 6,400 pixels, which is the threshold *T*_4_ applied for the ordering constraint.
3. Through the morphological operations, the process is able to break links between regions, allowing their identification. This occurs between regions 5 and 8 in the right, where two branches are overlapped. Without this breaking, both regions are labelled as a unique region and its matching with the corresponding regions in the left image, which are separated, is not possible.

### 4.2. Correspondence

At this stage we can compare the original stereo images displayed in Figures 1(a) and (b) with the labelled ones in Figure 9. It does not turn out to be difficult to determine the correspondences in this stereo pair based on our human observation.

In Table 1, the first column displays the number of labelled regions (trunks) in the left image and the second column the matched regions in the right image according to the human expert criterion.

The third and fourth columns display the *lk* and *rk* individual matches, as described in section 3.4. Finally, the fifth column shows the final decision in terms of successful decision (S) or unsuccessful (F) according to the criterion of the human observation through the matches established in the first and second columns.

From the results in Table 1, we can infer the following conclusions:

- We can see that regions labelled as 2, 1 and 3 in the left image match with regions 1, 5 and 3 respectively in the right image. Without the limitation of the ordering constraint with respect heights and areas of the regions, section 3.3, this constraint should be violated by the region 3 in the left image because the ordering with respect the couple 1 with 5 is not preserved. In this case, the ordering is applied only between regions 2, 1 in the left image and 1 and 5 in the right one. The heights and areas fulfil the requirements given in section 3.3. The ordering constraint is violated for the case of regions 19, 18 and 20 in the left image, which correspond to 18, 17 and 19, while the order is 18, 19 and 17. Based on the requirements in Section 3.3, they fulfil that the areas do not differ more than the 33% but they fails for the requirement that the areas must overpass the threshold *T*_4_, *i.e.*, in this case the ordering constraint is not applied.
- Occlusions: we have found a clear occlusion that has been correctly handled. The region 6 is visible in the left image and its corresponding match is occluded by 5 in the right image. Our approach does not find its match, as expected.
- Ambiguities: there are two types of ambiguities which arise inside the area of interest in the sample plot and outside this area. To the first case belongs the ambiguity between the region 13 in the left image and regions 12 and 7 in the right image. To the second case belong the regions 18 and 20 in the left image, where both have as preferred matches the regions 17 and 19. The first case is solved thanks to the application of the ordering constraint. Unfortunately, in the second case this constraint does not solve the ambiguity causing erroneous matches. Nevertheless, we still consider that its application is favourable because it works properly in the area of interest. Although this could be a limit for extending the area of interest.
- The percentage of successful correspondences in the stereo pair displayed in this paper is the 90%. On average, the percentage of success for the sixteen stereo pairs of images analyzed with similar characteristics is the 88.4%.

Overall, the combination of the two proposed processes, segmentation and correspondence, are suitable and robust enough for the kind of images analyzed.

## 5. Conclusions

In this paper we have proposed an automatic feature-based strategy for stereovision matching in omnidirectional images, acquired by a sensor equipped with two fish-eye lenses. We have designed two sequential processes: segmentation and correspondence. Several image processing techniques are applied for extracting regions (trunks) as features and their associated attributes. Based on these attributes and on the specific geometrical design of the sensor, we apply four well-known matching constraints in stereovision (*epipolarity, similarity, ordering* and *uniqueness*) for matching the regions during the correspondence process. The combination of these two processes makes the main finding of the paper for this kind of sensors and for the type of images analyzed.

The proposed approach is compared against the criterion applied by a human expert, which determines the correct matches. Finally, although the proposed stereovision matching strategy, based on fish eye lens systems, in this work has been primarily developed to improve the accuracy and reduce the costs in forest inventories, these techniques could be easily adapted for navigation purposes in forest with similar characteristics to the ones used in our experiments, *i.e.*, light environments. This kind of systems has been already used for robot navigation [36].

## Figures and Tables

**Table 1.** Results obtained using Hu moments for matching regions in both stereo pairs.

**Left image regions***L_i_*	**Corresponding right image regions** (*R_j_*)	***lk***	***rk***	**Final decision matching**
1	5	7	7	S
2	1	7	7	S
3	3	7	7	S
4	2	7	7	S
5	4	7	7	S
6	no match (hidden by 5)	0	0	S (unmatched)
7	6	7	7	S
8	8	4	5	S
9	10	6	7	S
10	7	7	1	S
11	9	7	7	S
12	11	7	3	S
13	12	1	7	S
14	13	7	7	S
15	14	7	7	S
16	15	7	7	S
17	16	7	7	S
18	17	2	4	F
19	18	5	6	S
20	19	4	3	F

## References

[1] Pita P.A. (1973). El Inventario en la Ordenación de Montes.

[2] Pardé J., Bouchon J. (1987). Dendrométrie.

[3] Mandallaz D., Ye R. (1999). Forest inventory with optimal two-phase, two-stage sampling schemes based on the anticipated variance. Can. J. Forest Res..

[4] Montes F., Hernández M.J., Cañellas I. (2005). A geostatistical aproach to cork production sampling estimation in Quercus suber L. forests. Can. J. Forest Res..

[5] Abraham S., Förstner W. (2005). Fish-eye-stereo calibration and epipolar rectification. Photogram. Remote Sens..

[6] Wulder M.A., Franklin S.E. (2003). Remote Sensing of Forest Environments: Concepts and Case Studies.

[7] Montes F., Ledo A., Rubio A., Pita P., Canellas I. (2009). Use of estereoscopic hemispherical images for forest inventories.

[8] Gregoire T.G. (1998). Design-based and model-based inference in survey sampling: apreciating the difference. Can. J. Forest Res..

[9] Barnard S., Fishler M. (1982). Computational Stereo. ACM Comput. Surv..

[10] Scharstein D., Szeliski A. (2002). Taxonomy and evaluation of dense two-frame stereo correspondence algorithms. Int. J. Comput. Vision.

[11] Tang L., Wu C., Chen Z. (2002). Image dense matching based on region growth with adaptive window. Pattern Recognit. Lett..

[12] Grimson W.E.L. (1985). Computational experiments with a feature-based stereo algorithm. IEEE Trans. Pattern Anal. Mach. Intell..

[13] Ruichek Y., Postaire J.G. (1996). A neural network algorithm for 3-D reconstruction from stereo pairs of linear images. Pattern Recognit. Lett..

[14] Medioni G., Nevatia R. (1985). Segment based stereo matching. Comput. Vision Graph. Image Process.

[15] Pajares G, Cruz J.M. (2006). Fuzzy cognitive maps for stereo matching. Pattern Recognit..

[16] Scaramuzza D., Criblez N., Martinelli A., Siegwart R., Laugier C., Siegwart R. (2008). Robust feature extraction and matching for omnidirectional images. Field and Service Robotics.

[17] McKinnon B., Baltes J., Klette R., Zunic J. (2004). Practical region-based matching for stereo vision.

[18] Marapane S.B., Trivedi M.M. (1989). Region-based stereo analysis for robotic applications. IEEE Trans. Syst..

[19] Wei Y., Quan L. Region-based progressive stereo matching.

[20] Chehata N., Jung F., Deseilligny M.P., Stamon G. (2003). A region-based matching approach for 3D-roof reconstruction from HR satellite stereo Pairs.

[21] Kaick O.V., Mori G. Automatic classification of outdoor images by region matching.

[22] Renninger L.W., Malik J. (2004). When is scene recognition just texture recognition?. Vision Res..

[23] Hu Q., Yang Z. Stereo matching based on local invariant region identification.

[24] Premaratne P., Safaei F. Feature based Stereo correspondence using Moment Invariant.

[25] Lopez M.A., Pla F. (2000). Dealing with segmentation errors in region-based stereo matching. Pattern Recognit..

[26] Wang Z.F., Zheng Z.G. (2008). A region based stereo matching algorithm using cooperative optimization.

[27] El Ansari M., Masmoudi L., Bensrhair A. (2007). A new regions matching for color stereo images. Pattern Recognit. Lett..

[28] Gonzalez R.C., Woods R.E. (2008). Digital Image Processing.

[29] Trias-Sanz R., Stamon G., Louchet J. (2008). Using colour, texture, and hierarchical segmentation for high-resolution remote sensing. ISPRS J. Photogram. Remote Sens..

[30] Bandzi P., Oravec M., Pavlovicova J. (2007). New statistics for texture classification based on gabor filters. Radioengineering.

[31] Herrera P.J., Pajares G., Guijarro M., Ruz J.J., de la Cruz J.M. (2009). Combination of attributes in stereovision matching for fish-eye lenses in forest analysis.

[32] Haralick R.M., Shapiro L.G. (1992). Computer and Robot Vision.

[33] Schwalbe E. Geometric modelling and calibration of fisheye lens camera systems.

[34] Elias R. (2007). Sparse view stereo matching. Pattern Recognit. Lett..

[35] Ogale A.S., Aloimonos Y. (2005). Shape and the stereo correspondence problem. Int. J. Comput. Vision.

[36] Shah S., Aggarwal J.K. (1997). Mobile robot navigation and scene modeling using stereo fish-eye lens system. Mach. Vision Appl..

